# FAMILY CAREGIVERS’ COMMUNICATION NEEDS AT THE END OF LIFE OF OLDER PATIENTS AT GERIATRIC FACILITIES

**DOI:** 10.1093/geroni/igz038.212

**Published:** 2019-11-08

**Authors:** Rinat Cohen, Gal Maydan, Shai Brill, Jiska Cohen-Mansfield

**Affiliations:** 1 Tel Aviv University, Tel Aviv, Israel, Israel; 2 Beit Rivka Geriatric Medical Center, Petach Tikva, Israel, Israel

## Abstract

Current literature on end-of-life communication (EOLC) between family caregivers (FCs) and health professionals (HPs) lacks reference to FCs’ communication needs and primarily addresses its formal aspects of communication such as offering advance directives. We explored FC’s communication needs and developed a questionnaire to evaluate the quality of EOLC. Interviews were conducted with 152 Israeli FCs of patients from nursing care, skilled nursing care, assisted ventilation, and dementia units within four facilities (nursing homes and geriatric medical centers). Most participants were women (61%), married (78%), and were children of the patients (77%), with a mean age of 57.5 (S.D.=12.01, range: 29-88). Qualitative analysis yielded several themes: FCs’ concerns about the availability and accessibility of all types of HPs, information needs (e.g., the need for regular updates initiated by HPs), FCs’ need for emotional support, and difficulties stemming from differences in language and culture. The need for improved communication in these spheres extended to all stages of hospitalization. Based on these needs, we developed a questionnaire to evaluate the quality of EOLC. Reliability was measured in a different sample, and ranged from Cronbach alpha of .916 (41 items; 41 FCs) to .937 (41 items with discrimination index greater than .3; 78 FCs). Factor analysis yielded factors similar to the themes that emerged from the qualitative analysis. The findings highlight aspects of EOLC between FCs and HPs which should be addressed and improved. Thus, this study is a crucial first step toward improving the quality of care at the end-of-life.

